# Time-dependent Inhibition of CYP2C8 and CYP2C19 by *Hedera helix* Extracts, A Traditional Respiratory Herbal Medicine

**DOI:** 10.3390/molecules22071241

**Published:** 2017-07-24

**Authors:** Shaheed Ur Rehman, In Sook Kim, Min Sun Choi, Seung Hyun Kim, Yonghui Zhang, Hye Hyun Yoo

**Affiliations:** 1 Department of Pharmacy, COMSATS Institute of Information Technology, Abbottabad 22060, Pakistan; dr.shaheedmarwat@yahoo.com; 2 Institute of Pharmaceutical Science and Technology and College of Pharmacy, Hanyang University, Ansan, Gyeonggi-do 15588, Korea; kis@hanyang.ac.kr (I.S.K.); chm2456@hanyang.ac.kr (M.S.C.); 3 College of Pharmacy, Yonsei Institute of Pharmaceutical Science, Yonsei University, Incheon 21983, Korea; kimsh11@yonsei.ac.kr; 4 School of Pharmacy, Tongji Medical College of Huazhong University of Science and Technology, Wuhan 430030, China; zhangyh@mails.tjmu.edu.cn

**Keywords:** *Hedera helix*, Araliaceae, CYP inhibition, human liver microsomes, herb–drug interaction

## Abstract

The extract of *Hedera helix L.* (Araliaceae), a well-known folk medicine, has been popularly used to treat respiratory problems, worldwide. It is very likely that this herbal extract is taken in combination with conventional drugs. The present study aimed to evaluate the effects of *H. helix* extract on cytochrome P450 (CYP) enzyme-mediated metabolism to predict the potential for herb–drug interactions. A cocktail probe assay was used to measure the inhibitory effect of CYP. *H. helix* extracts were incubated with pooled human liver microsomes or CYP isozymes with CYP-specific substrates, and the formation of specific metabolites was investigated to measure the inhibitory effects. *H. helix* showed significant inhibitory effects on CYP2C8, CYP2C19 and CYP2D6 in a concentration-dependent manner. In recombinant CYP2C8, CYP2C19 and CYP2D6 isozymes, the IC_50_ values of the extract were 0.08 ± 0.01, 0.58 ± 0.03 and 6.72 ± 0.22 mg/mL, respectively. Further investigation showed that *H. helix* extract has a positive time-dependent inhibition property on both CYP2C8 and CYP2C19 with IC_50_ shift value of 2.77 ± 0.12 and 6.31 ± 0.25, respectively. Based on this in vitro investigation, consumption of herbal medicines or dietary supplements containing *H. helix* extracts requires careful attention to avoid any CYP-based interactions.

## 1. Introduction

*Hedera helix* L. (Araliaceae), also known as common ivy or English ivy, has been traditionally used for the treatment of respiratory disorders [1]. The pharmacological data of *H. helix* extracts, including its bronchodilator, antibacterial, bronchospasmolytic, and expectorant effects, have supported its traditional use as a natural remedy for respiratory illness [2,3]. Currently, it is one of the top-selling herbal respiratory medicines in many countries worldwide, and it is popularly used for the treatment of cough and cough-related problems [4,5]. Bronchospasmolytic activity was exerted by hederacoside C, α-hederin, aglycone hederagenin, kaempferol and quercetin of *H. helix* extract [6]. Apigenin, kaempferol and quercetin significantly reduced the contraction of guinea-pig isolated ileum induced by prostaglandin E2 and leukotriene D4 [7]. The saponin from *H. helix* inhibited the terbutaline-stimulated internalization of the β2-adrenergic receptor in alveolar epithelial type-II cell line to explain its spasmolytic and β-mimetic effects [8,9]. Hederacoside C (HDC) is known as one of the primary constituents responsible for the therapeutic efficacy of *H. helix* extracts [10].

Unlike conventional drugs, herbal products are a complex mixture of bioactive constituents. As a result, their co-administration with prescription drugs may produce unexpected toxic or adverse consequences [11]. The main mechanisms underlying such interactions are via pharmacokinetic modulations such as inhibition or induction of drug-metabolizing enzymes and transporters. Among them, the inhibition of cytochrome P450 (CYP), a representative drug-metabolizing enzyme, is considered as one of the most frequent causes for herb–drug interactions [11,12]. Therefore, evaluating the inhibition of CYP enzyme activity by herbal and herb-derived medicine is vital to predict any possible pharmacokinetic interactions with conventional drugs and to characterize their safety profile.

Due to its properties as a respiratory remedy and a traditional herbal medicine, *H. helix* extracts are very likely to be used as an adjuvant to conventional drugs in treating various diseases accompanied by respiratory disorders. In this context, it is necessary to investigate and characterize the drug interactions with *H. helix* extracts to ensure safe use. It has been reported that liver enzymes are the major metabolizing enzymes to convert the principal bioactive constituents of *H. helix* to the secondary metabolites [13,14]. In two in vivo interaction studies [15,16], subcutaneously administered α-hederin influenced P450 enzymes in a dose-dependent manner, but no clinical relevance was expected from the results, as the IC_50_ values are high in comparison with its bioavailability [14]. However, to our knowledge, no previous studies have investigated how *H. helix* whole extracts affect CYP enzyme activity. Here, we examined the inhibitory effects of *H. helix* extract (as a whole) and its major bioactive constituent HDC on CYP450-mediated drug metabolism using human liver microsomes and individual recombinant CYP isozymes.

## 2. Results

### 2.1. CYP Inhibition Assay in Pooled Human Liver Microsomes

**Figure 1:**
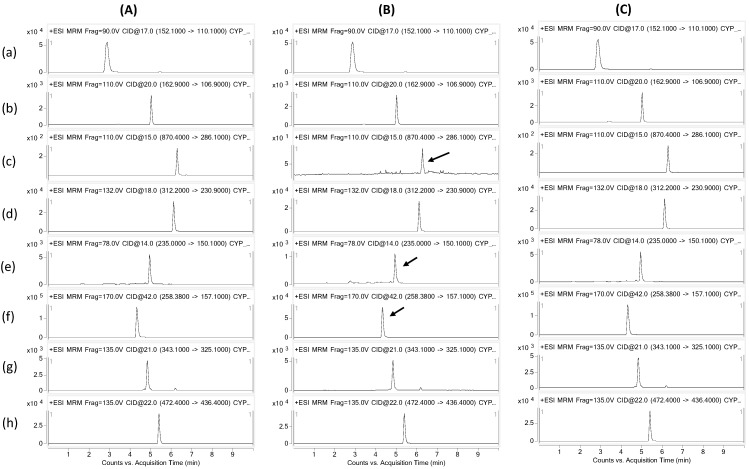
Representative multiple reaction monitoring (MRM) chromatograms of human liver microsome samples of, (**A**) control; (**B**) *H. helix* extract-treated, and (**C**) Hederacoside C (HDC)-treated. A fraction of human liver microsomal was incubated with the substrate mixture, in an NADPH-generating system, and *H. helix* extract (2.5 mg/mL) or HDC (100 µM) for 30 min and the cytochromes P450 (CYP)-specific metabolite formation was determined by LC-MS/MS. (a) Acetaminophen; (b) 7-OH coumarin; (c) 6-OH-paclitaxel (d) 4-OH-diclofenac; (e) 4-OH-mephenytoin; (f) dextrorphan; (g) 1-OH-madazolam; and (h) terfenadine (IS).

**Figure 2:**
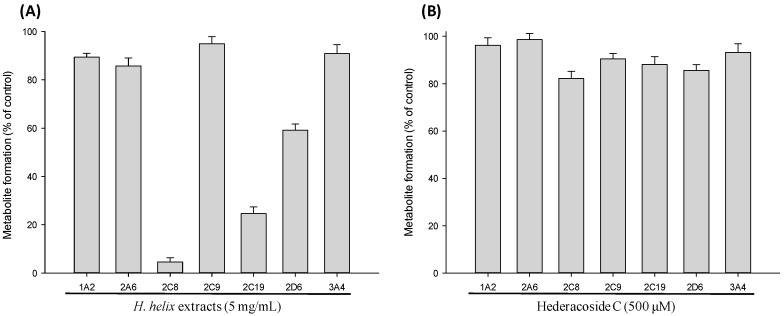

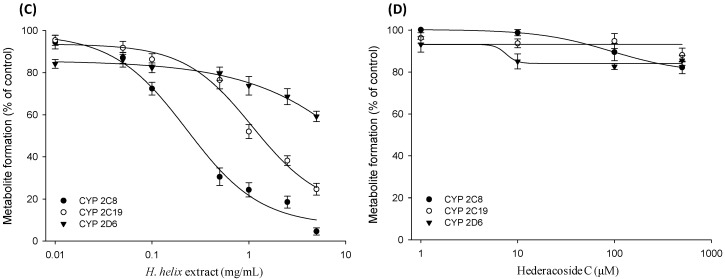
Effects of *H. helix* extract and hederacoside C on the CYP-specific metabolite formation in human liver microsomes. (**A**) *H. helix* extract (5 mg/mL); (**B**) Hederacoside C (500 μM); (**C**) Effects of *H. helix* extract on the metabolic activities of CYP2C8, CYP2C19, and CYP2D6; and (**D**) Effects of hederacoside C on the metabolic activities of CYP2C8, CYP2C19, and CYP2D6. Data was presented as the mean ± S.D. of the data obtained from two independent experiments.

We investigated the inhibitory effect of *H. helix* extract on CYP enzymes in pooled human liver microsomes. The CYP inhibition assay system was confirmed with the following well-known CYP-selective inhibitors: furafylline for CYP1A2, methoxsalen for CYP2A6, quercetin for CYP2C8, sulfaphenazole for CYP2C9, ticlopidine for CYP2C19, quinidine for CYP2D6, and ketoconazole for CYP3A4. Each of these inhibitors reduced the formation of each corresponding CYP-specific metabolite by >95%, indicating that the assay system was functioning well. The activities of seven CYP isozymes were tested with various concentrations of *H. helix* extracts, and the amount of metabolite produced at each concentration was measured. Figure 1 presents the representative multiple reaction monitoring (MRM) chromatograms of the control and *H. helix* extract/HDC-treated human liver microsome samples. Notably, *H. helix* extracts showed significant inhibitory activity against CYP2C8, CYP2C19, and CYP2D6 enzyme activity in a concentration-dependent manner (Figure 2A,C). The IC_50_ values of the extract against CYP2C8, CYP2C19 and CYP2D6 were 0.13 ± 0.01, 1.04 ± 0.06 and 7.41 ± 0.09 mg/mL, respectively. The inhibitory effects of the extracts on the other CYP isozymes were negligible at all the concentrations tested. As HDC is a known principal bioactive component of the *H. helix* extract, its effects on CYPs were also investigated. The resulting data showed slight inhibition of the CYP2C8 isozyme (18%) by HDC (Figure 2B,D), indicating that HDC is not primarily responsible for the CYP inhibition of *H. helix* extract.

### 2.2. CYP Inhibition Assay in cDNA-Expressed Recombinant CYP Isozymes

**Figure 3:**
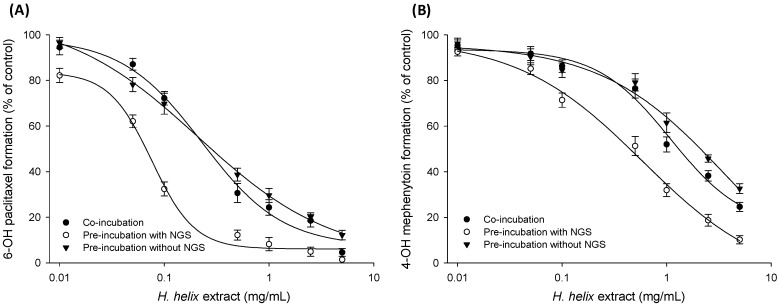

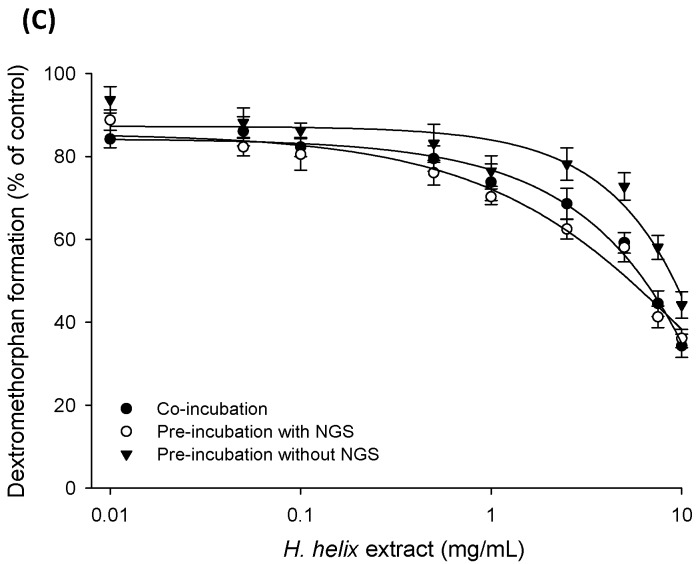
CYP-specific metabolite formation as the percent of control after co-incubation and pre-incubation of *H. helix* extracts with and without NADPH in c-DNA expressed CYP isozymes; (**A**) CYP2C8; (**B**) CYP2C19, and (**C**) CYP2D6. Data was presented as the mean ± S.D. of the data obtained from two independent experiments.

The results of the initial assessment with human liver microsomes revealed that *H. helix* extract significantly inhibited the enzyme activities of all three enzymes. Accordingly, the CYP inhibitory effect of *H. helix* extract was further investigated using the cDNA-expressed recombinant CYP isozymes for CYP2C8, CYP2C19, and CYP2D6. As a result, the inhibitory effect of the *H. helix* extract on CYP2C8, CYP2C19 and CYP2D6 was concentration-dependent, as shown in the assay with human liver microsomes (Figure 3). The IC_50_ values of the extract against CYP2C8, CYP2C19 and CYP2D6 were 0.08 ± 0.01, 0.58 ± 0.03 and 6.72 ± 0.22 mg/mL, respectively, which are lower than the corresponding levels in human liver microsomes. Additionally, the inhibitory effect of HDC against recombinant CYP isozymes was found to be negligible (data not shown); this finding was consistent with the data of human liver microsomes, confirming that the CYP inhibitory effect of *H. helix* extract was not attributed to HDC.

### 2.3. Time-Dependent Inhibition (TDI) and Mechanism-based Inactivation (MBI) Tests with Recombinant CYP2C8, CYP2C19 and CYP2D6 Isozymes

To elucidate whether the inhibition of CYP by *H. helix* extracts was time-dependent, the IC_50_ values derived from the co-incubation and pre-incubation assays were compared. Thus, the *H. helix* extract in recombinant CYP2C8, CYP2C19 and CYP2D6 isozymes was pre-incubated for 20 min with NADPH and the IC_50_ value shift for co-incubation was calculated. The inhibitory effects of *H. helix* extracts on CYP2C8 and CYP2C19 were more potent after pre-incubation (Figure 3), which suggested that the *H. helix* extract may act as a time-dependent inhibitor of CYP2C8 and CYP2C19. The IC_50_ shift for CYP2C8, CYP2C19 and CYP2D6 from the co-incubation assay to the pre-incubation assay was 3.01 ± 0.02, 1.88 ± 0.01 and 1.21 ± 0.06 mg/mL, respectively. The fold shift was calculated as the ratio of the IC_50_ of co-incubation (IC_50-co_) to that of the pre-incubation with NADPH IC_50-pre_ as follows:

IC_50_ shift-fold = IC_50-co/_IC_50-pre_

Compounds showing an IC_50_ shift of ≥1.5 can be classified as positive for TDI [17,18]. Based on this criterion, *H. helix* extracts may have a time-dependent inhibitory effect on CYP2C8 and CYP2C19. Another IC_50_ shift approach [19,20] was performed to identify the potential of MBI. For this, the IC_50_ shift from the pre-incubation assay in the absence and presence of NADPH was investigated (Figure 3). As a result, the IC_50_ shift for CYP2C8, CYP2C19 and CYP2D6 was 2.77 ± 0.12, 6.31 ± 0.25, and 1.38 ± 0.08 mg/mL, respectively. Compounds showing a ratio of IC_50_ shift of ≥4 can be classified as positive for MBI [19]. Thus, *H. helix* extracts were considered to be a mechanism-based inactivator on CYP2C19.

## 3. Discussion

CYP enzymes are vital to the metabolism of many conventional and herbal medicines. Some of these enzymes can be induced or inhibited by xenobiotics, resulting in clinically significant drug-drug or herb–drug interactions and causing unanticipated adverse reactions and/or therapeutic failure [17]. There are no standardized methods for detecting in vitro TDI, but they generally have in common a two-step process of pre-incubation with a test compound followed by the quantification of enzyme activity with a probe substrate. The MBI represents the TDI, where the inhibitory effects are not only time-dependent but they also require metabolism by the enzyme that is ultimately inactivated [20]. Thus, time-dependent enzyme inhibitors display an increasing degree of enzyme inhibition with an increased pre-incubation time with the enzyme. For CYP enzymes, a source of NADPH cofactor is often required to generate MBI. The enzymatic process leading to MBI is irreversible, and catalytic activity cannot be restored. However, the enzyme inactivation occurring in the pre-incubation step must be distinguished from reversible inhibition by comparison to a suitable control (i.e., without NADPH) incubation, to predict NADPH-independent metabolism as well as enzyme degradation. The presence of NADPH in the absence of a substrate may accelerate enzyme activity loss (possibly due to generation of reactive oxygen species) or exert a stabilizing effect, depending on the enzyme and assay conditions [21]. Compared to reversible CYP inhibition, drug-induced MBI (a quasi-irreversible or irreversible inhibition) of CYP enzymes poses a greater safety concern because of the increased risk of pharmacokinetic interactions upon multiple dosing and the sustained duration of such interactions even after the termination of such entities [22]. Characterization of the inactivation property of CYP is essential for predictions of the drug–drug or herb–drug interaction potential of TDI-positive medicines [20]. Recently, guidelines from regulatory agencies such as the United States Food and Drug Administration [23] and pharmaceutical companies [24] have recognized the importance of mitigating drug interaction risks, particularly with respect to CYP TDI/MBI. In pre-clinical drug discovery, the in vitro assessment of PK interactions through TDI is routinely conducted for lead optimization [22].

As herbal medicines (extracts) are a complex mixture formed by various chemical entities [25], some of these constituents possibly contain the functional group responsible for the MBI of CYPs [20,26]. Many studies have reported the MBI of human CYPs by constituents isolated from traditional herbal medicines [27,28,29]. For the TDI assessment with *H. helix* extracts, IC_50_ shifts were calculated by comparing IC_50_ vales from the co-incubation assay to the pre-incubation assay [17], which showed positive results (≥1.5) for CYP2C8 and CYP2C19. As for MBI, the IC_50_ shift was demonstrated from the ratio of the IC_50_ value from the pre-incubation assay in the absence of NADPH to that from pre-incubation in its presence. Based on the resulting data, the *H. helix* extracts were considered positive for CYP2C19 (IC_50_ shift ≥4) [19]. Thus, our in vitro results indicated that *H. helix* shows TDI effects for CYP2C8 and CYP2C19, but it has a positive MBI response only for CYP2C19. CYP2D6 inhibition was slightly effected during this study, indicating that *H. helix* has minimal effects as TDI or MBI. 

Based on our findings, there is a potential for herb–drug interactions between the *H. helix* extract and commonly prescribed conventional medicines that are CYP2C8, CYP2C19 and CYP2D6 substrates. The most sensitive in vivo substrates for CYP2C8 include repaglinide, rosiglitazone and paclitaxel, while quercetin and montelukast are the inhibitors and moderate sensitive substrates for CYP2C8 [23]. Lansoprazole, omeprazole, esoprazole, and *S*-mephenytoin are the in vivo sensitive substrates for CYP2C19, while dextromethorphan is a substrate for CYP2D6 [23]. In particular, paclitaxel and *S*-mephenytoin are known to have a narrow therapeutic range, while the drugs used for respiratory illness like dextromethorphan and montelokast are quite sensitive here. Therefore, the concomitant use of these drugs with *H. helix* extracts may result in clinically significant drug interactions and requires careful attention. The dosage of *H. helix* preparations is discussed contradictorily in the literature. In a controlled study, the efficacy was shown with low dosages (approximately 300 mg herbal substance), while in the market there are preparations with daily dosages up to approximately 1000 mg herbal substance (Committee on Herbal Medicinal Products, European Medicines Agency) [4,10,14]. The clinical pharmacokinetic data on *H. helix* extracts is unavailable at present. Although the absorption of *H. helix* extract from the gastrointestinal tract is shown to be somewhat inadequate for some constituents like hederacoside C and α-hederin [10,14], if the extract is assumed as a single compound, its plasma concentration could reach up around 1 mg/mL. Therefore, it might have clinically close relevance for CYP-mediated herb–drug interactions, at least for CYP2C8 and CYP2C19, considering its maximum daily dosages. 

## 4. Materials and Methods

### 4.1. Chemicals and Reagents

**Figure 4:**
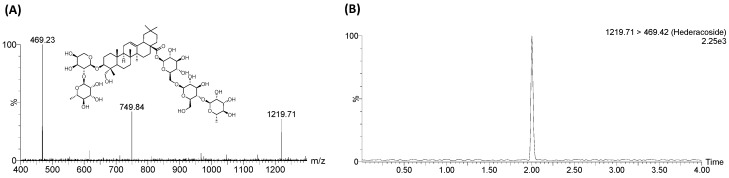
Hederacoside C, (**A**) product ion mass spectra of the [M − H]^−^ ions of HDC, and (**B**) MRM chromatograms of HDC in *H. helix* extracts.

HDC (>98.0%) and *H. helix* extract were obtained from the Lab. of Pharmacognosy, College of Pharmacy, Yonsei University (Incheon, Korea). The extract was prepared by extracting the pulverized ivy leaves with 30% ethanol for 1 h using sonication. A voucher specimen of *H. helix* extract (HY-2016-01-05) was deposited at the Herbarium of the College of Pharmacy, Hanyang University, Ansan, Korea. *H. helix* extract contains 8.2% of HDC. The content of HDC was determined by liquid chromatography–tandem mass spectrometry (LC-MS/MS) [10] and the representative chromatogram is shown in Figure 4. Pooled human liver microsomes and recombinant CYP2C8, CYP2C19, and CYP2D6 isozymes were purchased from BD Gentest (Woburn, MA, USA). Glucose-6-phosphate, β-NADP+, glucose-6-phosphate dehydrogenase, coumarin, phenacetin, diclofenac, midazolam, mephenytoin, dextromethorphan, ketoconazole, and terfenadine were purchased from Sigma Chemical Co. (Saint Louis, MO, USA). All other solvents used were of HPLC grade and were obtained from J. T. Baker (Phillipsburg, NJ, USA). Distilled water was prepared using a Milli-Q purification system (Millipore, Billerica, MA, USA). All standard solutions and mobile phases were passed through a 0.22 µm membrane filter before use.

### 4.2. CYP Inhibition Assay

CYP inhibition assays with human liver microsomes were performed for *H. helix* extract at various concentrations (0.01, 0.05, 0.1, 0.5, 1.0, 2.5 and 5 mg/mL) and HDC (1, 10, 100, and 500 µM) according to the method used in our previous study [30] (For the determination of the IC_50_ value for CYP2D6, the concentration range of *H. helix* extract was extended up to 10 mg/mL). The reaction mixtures consisted of human liver microsomes at a concentration of 0.5 mg/mL; *H. helix* extract or HDC standard solution at various concentrations; an NADPH-generating system (NGS, containing 0.1 M glucose-6-phosphate, 10 mg/mL β-NADP+, and glucose-6-phosphate dehydrogenase) at 1.0 U/mL; and probe substrates (the final concentrations were at ~Km, which were 40 µM phenacetin, 2.5 µM coumarin, 5 µM dextromethorphan, 10 µM diclofenac, 160 µM mephenytoin, 10 µM paclitaxel, and 2.5 µM midazolam) in 200 µL of 0.1 M potassium phosphate buffer (pH 7.4). The reaction mixture was pre-incubated at 37 °C for 5 min without NGS and then further incubated for 30 min with NGS in a water bath. Ketoconazole (5 µM), furafylline (10 µM), methoxsalen (10 µM), sulfaphenazole (50 µM), ticlopidine (20 µM), quercetin (30 µM), and quinidine (50 µM), which are all CYP specific inhibitors, were tested as positive controls to confirm substrate selectivity for various CYP isoforms. After the incubation, the reaction was stopped by adding 400 µL of 0.1% acetic acid containing internal standard (0.16 µM terfenadine). For the further investigation of specific CYP isozyme inhibition, 12.5 pmol isozyme (CYP 2C8, 2C19, and 2D6) were used instead of human liver microsomes, and the corresponding specific substrates were added to the reaction mixture (10 µM paclitaxel, 160 µM mephenytoin, and 5 µM dextromethorphan, respectively), following the procedure as described before. For time-dependent inhibition, the reaction mixtures consisting of recombinant CYP2C8/CYP2C19/CYP2D6, and NGS in a 0.1 M potassium phosphate buffer, was pre-incubated with *H. helix* extract for zero (co-incubation) and 20 min, followed by the addition of the corresponding substrates (i.e., paclitaxel (CYP2C8) or mephenytoin (CYP2C19) or dextromethorphan (CYP2D6)), and further incubated for 30 min in water bath (followed by rest of the procedure, as described). For the determination of IC_50_ values for mechanism-based inactivation, the recombinant CYP isozyme was pre-incubated for 30 min in the presence and absence of NGS [18].

### 4.3. Sample Preparation

For sample preparation, the incubation mixtures were passed through activated Sep-Pak C18 cartridges (96-well OASIS HLB Extraction Cartridge, Waters, Milford, MA, USA). The cartridges were first eluted with methanol (1 mL) and 0.1% acetic acid (1 mL). After loading the sample, the cartridges were washed twice with 0.1% acetic acid (2 mL) and finally eluted with 1 mL of methanol. The eluate was evaporated under nitrogen gas, and the residue was reconstituted in the mobile phase (100 µL, 0.1% formic acid in an 85/15 mixture of water/acetonitrile, *v*/*v*). A 5 µL aliquot was injected into the LC-MS/MS system.

### 4.4. LC-MS/MS Analysis

The LC-MS/MS system consisted of an Agilent 1260 series binary pump HPLC system and an Agilent 6460 Triple Quadrupole mass spectrometer (Agilent Technologies, Palo Alto, CA, USA) which was equipped with the source of an electrospray ionization (ESI). Chromatographic separation was conducted by using a Fortis-C_8_ column (2.1 mm × 100 mm, 5 µm; Fortis Technologies Ltd., Cheshire, UK), and the column temperature was maintained at 40 °C. The composition of the HPLC mobile phases were (A) 0.1% formic acid in distilled water and (B) 90% acetonitrile in the solvent A. A gradient program was used at a flow rate of 0.2 mL/min; the composition of solvent B was initially set at 15%, then gradually increased to 85% (15−85%) over 3 min, and maintained for 1.5 min (85%), and finally re-equilibrium for 3.5 min (85−15%). The mass spectrometer was operated in the positive ion mode, with MRM. The precursor–product ion pairs (Q1/Q3) used in MRM mode are shown in Table 1.

### 4.5. Statistics

Data was presented as the mean ± S.D. of the data obtained from two independent experiments. The IC_50_ value was calculated with the metabolite-formation values based on the four parameters logistic regression using Sigmaplot v12.5 (Systat Software, Inc., WPCubed GmbH, Munich, Germany) and GraphPad Prism v7.1 (GraphPad Software, Inc., La Jolla, CA, USA).

## 5. Conclusions

The *H. helix* extract is a popularly used traditional medicine as well as one of the top-selling herbal medicines for respiratory disorders in many countries. Despite its high possibility of co-administration with other prescribed drugs, no studies have investigated its inhibitory potential on CYP enzyme activities. In the present study, we reported CYP enzyme inhibition by *H. helix* extracts for the first time. *H. helix* extract inhibited the enzyme activities of CYP2C8, CYP2C19 and CYP2D6 in a concentration-dependent manner, while it showed time-dependent inhibition for CYP2C8 and CYP2C19. Furthermore, our results suggested that *H. helix* reacts as a mechanism-based inactivator for CYP2C19 (IC_50_ shift ≥4). Based on these findings, consuming herbal medicines or dietary supplements containing *H. helix* extracts requires careful attention and further in vivo studies should investigate CYP-mediated interactions with *H. helix* to verify the potential of herb–drug interactions and to establish proper recommendations for the safe therapeutic use of *H. helix*.

## Figures and Tables

**Table 1:** Information on the probe substrates and their corresponding CYP-specific metabolites used in this study.

P450 Isozyme	PROBE Substrate	Substrate Conc. (µM)	Metabolite Monitored	Precursor-Ion (*m*/*z*)	Daughter-Ion (*m*/*z*)
**CYP**1A2	Phenacetin	40	Acetaminophen	152.1	110.1
**CYP**2A6	Coumarin	2.5	7-OH-coumarin	162.9	106.9
**CYP**2C8	Paclitaxel	10	6-OH-paclitaxel	870.4	286.1
**CYP**2C9	Diclofenac	10	4-OH-diclofenac	312.2	230.9
**CYP**2C19	Mephenytoin	160	4-OH-mephenytoin	235.0	150.1
**CYP**2D6	Dextromethorphan	5	Dextrorphan	258.3	157.1
**CYP**3A4	Midazolam	2.5	1-OH-midazolam	343.1	325.1
**Internal Standard**		-	Terfenadine	472.4	436.4
